# Quantitative evaluation of hybridization and the impact on biodiversity conservation

**DOI:** 10.1002/ece3.2595

**Published:** 2016-12-20

**Authors:** Anna M. van Wyk, Desiré L. Dalton, Sean Hoban, Michael W. Bruford, Isa‐Rita M. Russo, Coral Birss, Paul Grobler, Bettine Janse van Vuuren, Antoinette Kotzé

**Affiliations:** ^1^ National Zoological Gardens of South AfricaPretoriaSouth Africa; ^2^ Genetics DepartmentUniversity of the Free StateBloemfonteinSouth Africa; ^3^ Department of Life Sciences and BiotechnologyUniversity of FerraraFerraraItaly; ^4^ The Morton ArboretumLisleILUSA; ^5^ National Institute for Mathematical and Biological Synthesis (NIMBioS)University of TennesseeKnoxvilleTNUSA; ^6^ Cardiff School of BiosciencesCardiff UniversityCardiffUK; ^7^ CapeNatureStellenboschSouth Africa; ^8^ Molecular Zoology LaboratoryDepartment of ZoologyUniversity of JohannesburgAuckland ParkSouth Africa

**Keywords:** blesbok, bontebok, hybridization, HYBRIDLAB, NEWHYBRIDS, STRUCTURE

## Abstract

Anthropogenic hybridization is an increasing conservation threat worldwide. In South Africa, recent hybridization is threatening numerous ungulate taxa. For example, the genetic integrity of the near‐threatened bontebok (*Damaliscus pygargus pygargus*) is threatened by hybridization with the more common blesbok (*D. p. phillipsi*). Identifying nonadmixed parental and admixed individuals is challenging based on the morphological traits alone; however, molecular analyses may allow for accurate detection. Once hybrids are identified, population simulation software may assist in determining the optimal conservation management strategy, although quantitative evaluation of hybrid management is rarely performed. In this study, our objectives were to describe species‐wide and localized rates of hybridization in nearly 3,000 individuals based on 12 microsatellite loci, quantify the accuracy of hybrid assignment software (STRUCTURE and NEWHYBRIDS), and determine an optimal threshold of bontebok ancestry for management purposes. According to multiple methods, we identified 2,051 bontebok, 657 hybrids, and 29 blesbok. More than two‐thirds of locations contained at least some hybrid individuals, with populations varying in the degree of introgression. HYBRIDLAB was used to simulate four generations of coexistence between bontebok and blesbok, and to optimize a threshold of ancestry, where most hybrids will be detected and removed, and the fewest nonadmixed bontebok individuals misclassified as hybrids. Overall, a threshold *Q*‐value (admixture coefficient) of 0.90 would remove 94% of hybrid animals, while a threshold of 0.95 would remove 98% of hybrid animals but also 8% of nonadmixed bontebok. To this end, a threshold of 0.90 was identified as optimal and has since been implemented in formal policy by a provincial nature conservation agency. Due to widespread hybridization, effective conservation plans should be established and enforced to conserve native populations that are genetically unique.

## 1 Introduction

One of the goals of conservation is to conserve current biodiversity as well as the ecological circumstances and evolutionary processes that support it. Increasingly, biodiversity is being adversely affected by human actions. Anthropogenic hybridization is increasing worldwide and is a threat to the conservation of species (Todesco et al., 2016). This human‐mediated hybridization may occur due to the changes in the abundance and distribution of species, the removal of barriers that cause isolated or restricted species to expand, and/or the uncontrolled diffusion with domestic species (Allendorf, Leary, Spruell, & Wenburg, 2001; Allendorf, Luikart, & Aitken, 2013; Rhymer & Simberloff, 1996). Molecular markers have been successfully used over the past decades to identify the rates of hybridization with high accuracy and low cost (e.g., Costa et al., 2013; Cullingham et al., 2011; Stephens, Wilton, Fleming, & Berry, 2015). Managing interspecific hybridization, after it is identified, is a more difficult task (Laikre, Schwartz, Waples, & Ryman, 2010; Piett, Hager, & Gerrard, 2015). As of yet, molecular marker data are rarely integrated with management decisions in a quantitative, predictive framework (Hoban et al., 2013).

In South Africa, wildlife species are extensively translocated outside of their historic distribution ranges onto private land as a part of wildlife management and commercial breeding (Spear & Chown, 2009). Due to private ownership of wildlife in South Africa, there is frequent trade in commercially profitable species which has led to the occurrence of multiple species on the same property outside their natural ranges. Thus, the incidence of hybridization has increased due to the scarcity of conspecific mates (Vaz Pinto, Beja, Ferrand, & Godinho, 2016) and loss of reproductive barriers between previously isolated evolutionary lineages. The potential negative impacts of hybridization are rapidly becoming a concern for South African conservation agencies. Hybridization may disrupt adaptive gene complexes or may result in genetic incompatibilities. Hybridization is known to reduce fitness in at least one species pair in South Africa, namely kudu (*Tragelaphus strepsiceros*) and nyala (*T. angasii*) (Dalton et al., 2014). An additional threat of hybridization is complete swamping, as in the rediscovered Giant sable antelope (*Hippotragus niger variani*), where natural hybridization with roan antelope (*H. equinus*) was proceeding rapidly and would have led to a complete hybrid swarm without intervention (Vaz Pinto et al., 2016). Consequences of anthropogenic hybridization include reduced fertility in the rare taxon, genetic swamping, or assimilation (Levin, Francisco‐Ortega, & Jansen, 1996; Malukiewicz et al., 2014), which may lead to eventual extinction (Wolf, Takebayashi, & Rieseberg, 2001). In some cases, hybrids persist at low levels, while in other cases hybrid swarms effectively replace the original species (Allendorf et al., 2001). Hybridization rates can increase over time, sometimes very rapidly (Huxel, 1999; Schwartz, Luikart, & Waples, 2007). A recent review of 62 anthropogenic hybridization cases found that nearly all had identified negative consequences, especially for mammals (Piett et al., 2015).

The fate of hybrid animals is controversial. Management approaches could include culling all hybrid animals, isolation of hybrid herds, certification of nonadmixed herds, planned breeding, and/or legislation to restrict the movement of hybrids and nonadmixed species to prevent future hybridization events (Grobler et al., 2011). These interventions should be considered if introgression is widespread, with hybrid offspring being fertile where one or both taxa are threatened (Allendorf & Luikart, 2007; Laikre et al., 2010; Piett et al., 2015; Schwartz et al., 2007). Hybridization is currently threatening the genetic integrity of numerous ungulate taxa in South Africa such as blue wildebeest (*Connochaetes taurinus*) and black wildebeest (*C. gnou*) (Grobler et al., 2011), black‐faced impala (*Aepyceros melampus petersi*) and common impala (*A. melampus*) (Green & Rothstein, 1998), Grevy's zebra (*Equus grevyi*) and plains zebra (*E. quagga*) (Cordingley et al., 2009), and bontebok (*Damaliscus pygargus pygargus*) and blesbok (*D. p. phillipsi*) (Lloyd & David, 2008).

**Figure 1:**
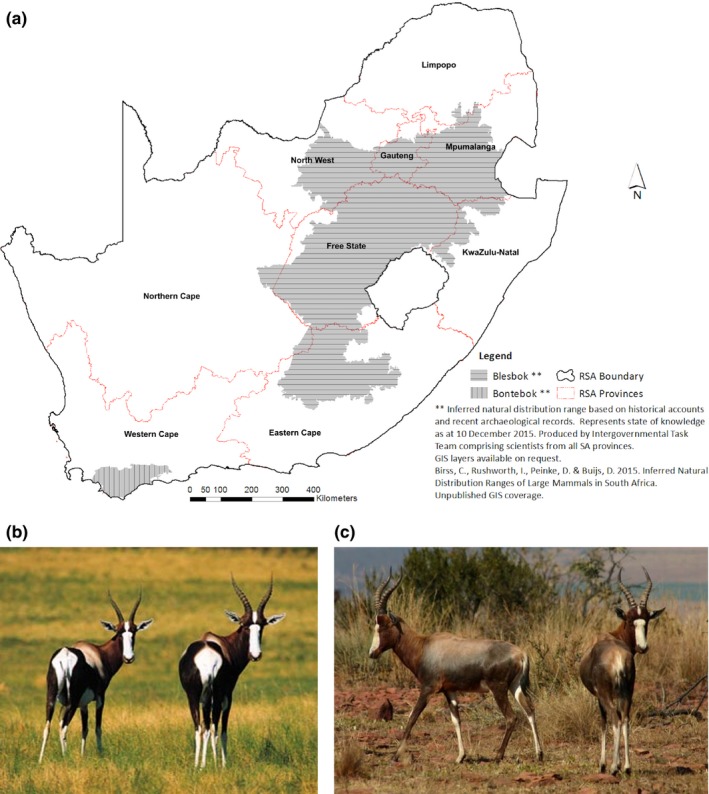
(a) Map indicating provinces in South Africa and historical distribution ranges of the bontebok and blesbok. (b) Photographic representation of a pure bontebok. (c) Photographic representation of a pure blesbok

This article will focus on two subspecies of *Damaliscus pygargus*, nl. bontebok (*D. p. pygargus*) and blesbok (*D. p. phillipsi*). These two taxa are South African endemics (Vrba, 1979) with the common ancestor being historically distributed from the southwestern Cape to the southern boundary of Zimbabwe (Vrba, 1979). Fossil evidence indicates that past climatic and habitat changes resulted in the separation of *D. pygargus* into two allopatric groups (Skead, 1980; Skinner & Smithers, 1990), which are now classified as separate subspecies (Essop, Lloyd, Van, & Harley, 1991; Van der Walt, Nel, & Hoelzel, 2001). Historically, the blesbok occurred mostly in the grassland biomes in Gauteng, Eastern Cape, Mpumalanga, and the Free State Provinces (Figure 1, Skinner & Smithers, 1990). The bontebok had a more restricted distribution to the low‐lying, grassy coastal plains within the fynbos biome of the Western Cape Province, where the population has declined and was driven to near extinction due to hunting and human intrusion in the 1800s (Van der Merwe, 1986). The two subspecies had non‐overlapping ranges within different ecosystems (Figure 1). Translocations to wildlife farms and reserves outside the former distribution ranges have brought the two subspecies in artificial, secondary contact. These events have led to documented hybridization between the two subspecies (Van der Walt et al., 2001; Van Wyk, Kotzé, Randi, & Dalton, 2013).

The primary threat to bontebok in the Western Cape Province is low availability of suitable habitat, thus limiting population expansion. Previous studies revealed low genetic diversity (Van Wyk et al., 2013), population fragmentation, and the deliberate and/or accidental hybridization with blesbok. The total number of bontebok in South Africa is estimated between 6,500 and 7,000 animals with less than 1,000 individuals occurring within its former distribution range (Unpublished, CapeNature, 2014). The bontebok is listed as near‐threatened on the International Union for Conservation of Nature (IUCN) Red List of Threatened Species.

A photographic test to distinguish between hybrids, nonadmixed bontebok, and blesbok was developed by Fabricius, van Hensbergen, and Zucchini (1989). The characteristics chosen as criteria for distinguishing between bontebok and blesbok were described by Bigalke (1955), emphasizing the importance of the white buttocks, upper legs, and belly. The photographic test has some shortfalls that require human interpretation, and in certain cases, hybrids could not be identified (especially in the F2 and subsequent generations). Notwithstanding, bontebok purity certificates were issued for tested populations based on photography. Due to the difficulties in characterizing hybrids based on morphological characteristics, a more accurate DNA test using a model‐based Bayesian approach was developed that could be used to identify nonadmixed individuals and hybrids (Van Wyk et al., 2013). The extent of hybridization across the species range is as yet unknown but must be determined prior to conservation intervention. The Western Cape Provincial Conservation Agency, CapeNature, has been mandated to develop the Bontebok Conservation, Translocation and Utilization Policy (BCTUP, 2014) as a regulatory mechanism to direct the implementation of a genetic purity test for bontebok prior to any approval of translocations.

Sophisticated algorithms for molecular marker data have been used extensively to identify hybrids. The selection of a threshold *Q*‐value (hybridization or admixture index from clustering algorithms like STRUCTURE) can affect the classification of nonadmixed animals and hybrids. Performance of hybrid identification should be a balance of both accuracy and efficiency (Vähä & Primmer, 2006), reflecting an offset between errors of inclusion (identifying all hybrids at the expense of including some nonadmixed individuals) and omission (omitting hybrids to ensure that nonadmixed individuals are not mistaken as hybrids). In the literature, *Q*‐values ranging from 0.7 to 0.99 are commonly used (Hoban, McCleary, Schlarbaum, Anagnostakis, & Romero‐Severson, 2012; Lepais et al., 2009; Sanz, Araguas, Fernández, Vera, & García‐Marín, 2008; Valbuena‐Carabaña, González‐Martínez, Hardy, & Gil, 2007). There is a trade‐off between accuracy and efficiency and between focusing on the nonadmixed species or on hybrids. The optimal *Q*‐value depends on the application of the test and the target species. This value can be determined via simulations (Cullingham et al., 2011; Lepais et al., 2009). Simulations can then also be used to determine the consequence of culling animals deemed to be hybrids (Hoban, 2014; Huxel, 1999).

In this study, we describe species‐wide hybridization rates using close to 3,000 bontebok blood, tissue, or hair samples. Our aims are to (1) accurately describe species‐wide and local hybridization rates and assess the use of different software for identifying hybrids, (2) determine a threshold for the classification of hybrid and nonadmixed animals using simulated data, and (3) quantify how many animals would be removed based on the selected threshold.

## 2 Materials and methods

### 2.1 Sample collection

The study formed part of an ongoing registered project entitled “Detection of hybridization and determination of the level of genetic diversity in South African antelope: blesbok and bontebok.” The project was approved by SANParks in 2009, and ethical approval was obtained from the Research, Ethics and Scientific Committee of the National Zoological Gardens of South Africa (project P10/31). Blood, tissue and hair samples were collected from bontebok (within and outside their historical distribution range) that had a documented history of origin and known isolation from blesbok. A total of 76 nonadmixed bontebok (Bontebok and De Hoop National Parks [Western Cape]) were collected as reference material. In addition, a total of 70 nonadmixed blesbok blood, tissue, and hair samples (Northern Cape and Free State Provinces) were collected from populations that were isolated from bontebok within and outside their historic distribution range. These animals were taken as representing animals of certain provenance or purity. Samples of unknown purity (*n *= 2,832) were collected to detect hybrids on private wildlife ranches in South Africa (Northern Cape, Free State, Eastern Cape, Western Cape, Gauteng, North West Provinces). Thus, the whole dataset (*n *= 2,978) consisted of nonadmixed bontebok, nonadmixed blesbok, and samples of unknown purity (grouped according to the collection locality).

### 2.2 Microsatellite genotyping and genetic diversity

DNA was extracted using the Qiagen DNeasy^®^ Blood and Tissue Kit (GmbH, Germany), following the extraction protocol as outlined by the manufacturer. Samples were genotyped at 12 microsatellite loci: BB10, BM1824, BB05, BB08, BB03, BB04, OARCP26, ETH10, BM203, BB20, BB22, and BM2113 as described in Van Wyk et al. (2013). MICRO‐CHECKER (Van Oosterhout, Hutchinson, Wills, & Shipley, 2004) was used to detect possible genotyping errors, allele dropout, and nonamplified alleles (null alleles). This software package can estimate the frequency of null alleles and adjust the dataset to correct for genotyping errors. Mean number of alleles per locus (A), observed heterozygosity (Ho), expected heterozygosity (He), and deviations from Hardy–Weinberg (HW) proportions were calculated for the two reference species (bontebok and blesbok) using MS Toolkit (Park, 2001), the R package adegenet (Jombart, 2008; Jombart & Ahmed, 2011) and GenAlEx 6.5 (Peakall & Smouse, 2006, 2012). Linkage disequilibrium between pairs of microsatellite loci within each species and locus was evaluated using Genepop 4.0 (Raymond & Rousset, 1995). Associated probability values were corrected for multiple comparisons using Bonferroni adjustment for a significance level of 0.05.

### 2.3 Admixture analysis

Identification and classification of hybrid individuals were conducted using four different methods: principal component analysis (PCA), assignment of individuals based on species‐specific alleles, and two Bayesian clustering software programs. A PCA of a pairwise, individual‐by‐individual, covariance matrix was calculated using multivariate ordination methods without spatial components in GenAlEx 6.5 (Peakall & Smouse, 2006, 2012) and adegenet (Jombart, 2008; Jombart & Ahmed, 2011) to examine the relationships among individuals without prior grouping. This method was used to provide information with regard to the divergence among groups and identify individuals that clustered separately from nonadmixed populations, thus indicating hybrid origin.

Assignment of individuals was also determined using species‐specific alleles (e.g., individuals with blesbok alleles) as an alternative to model‐based clustering methods (Metcalf, Siegle, & Martin, 2008). We examined allele frequency plots for bontebok and blesbok, based on individuals assigned to their respective species with admixture coefficient values *Q* > 0.97 (identified in STRUCTURE). We identified blesbok‐specific and bontebok‐specific alleles if the allele was (a) at appreciable frequency in species A (>0.05), (b) absent or nearly so from species B (<0.005), and (c) distant by at least three base pairs from a known allele in species B. We calculated the number of hybrids, blesbok, and bontebok that included at least one blesbok‐specific allele, thus indicating their hybrid status. We also found some alleles that are fixed (frequency ~ 1.0) in bontebok, and so we counted the number of hybrids, blesbok, and bontebok that included all of these alleles.

Lastly, two Bayesian clustering software programs, STRUCTURE version 2.3.4 (Hubisz, Falush, Stephens, & Pritchard, 2009; Pritchard, Stephens, & Donnelly, 2000) and NEWHYBRIDS versions 1.1 (Anderson & Thompson, 2002), were used to determine the admixture status of the individuals. To quantitatively compare the performance of the two programs, various parameter settings, models, and run lengths were tested. The effects of the different combinations were assessed by comparing the membership coefficient value (*Q*‐value; the probability of an individual belonging to a cluster; an intermediate value for both clusters is evidence of recent genetic admixture) for each individual as well as overall assignment rates to each of the hybrid categories, to determine the degree to which these settings influenced our results. STRUCTURE with the number of a priori clusters (K) set to 2 (under the assumption that both species contribute to the gene pool of the individuals) was used to estimate the admixture status for each individual. STRUCTURE is a model‐based method that assumes two separate gene pools that had recent contact. Analysis was thus performed using both “uncorrelated” and “correlated” frequencies. All runs were performed assuming the admixture model that allows for hybrid offspring. For each analysis, to determine the appropriate Markov chain Monte Carlo length and burn‐in, we tested “short” (total length* *= 1e5, burn‐in* *= 1e4), “long” (total length* *= 1.2e6, burn‐in* *= 2e5), and “very long” (total length* *= 2.4e6, burn‐in* *= 4e5) runs. We performed a minimum of five replicates under each setting. For all runs, the genetic ancestry of the “reference” sets (option LocPrior) was not provided as prior information to STRUCTURE. NEWHYBRIDS version 1.1 (Anderson & Thompson, 2002) assumes two clusters with recent (up to several generations) admixture. Each individual is assigned a probability of belonging to one of the six possible categories: nonadmixed species A, nonadmixed species B, F1, F2 (intercross), backcross to species A and backcross to species B. The probabilities across all six categories sum to 1. The program takes into account prior information on allele frequencies and the amount of admixture. In the absence of information of these two factors, we used Jeffrey's prior (Gelman, 2009). However, to rule out the possibility of bias due to low frequencies of alleles, the uniform prior analysis was also run. We performed a minimum of three replicate runs for each prior, for both moderate (burn‐in of 1e4, total run length of 1e5) and long (burn‐in of 2e5, total run length of 1.2e6) runs.

### 2.4 Simulation analysis

Simulated data were created using HYBRIDLAB (Nielsen, Bach, & Kotlicki, 2006). This program creates hybrids from two parental population allele frequency pools as determined from frequencies calculated from our reference dataset. We created a test dataset of 4,000 simulated individuals in which the ancestry of all individuals is known. The test dataset consisted of 500 each of nonadmixed bontebok, nonadmixed blesbok, F1 hybrids, F2 hybrids (backcross to bontebok or blesbok or F1), backcross to bontebok, backcross to blesbok, double backcross to bontebok and double backcross to blesbok. The simulated dataset was subsequently analyzed with STRUCTURE and NEWHYBRIDS, with settings as described above to calculate admixture values (as in Vähä & Primmer, 2006; Cullingham et al., 2011; Hoban et al., 2012). The advantage of using simulated data is that we know which individuals belong to each class, so we are able to assess whether Bayesian assignments are correct, and to quantify how well these programs assign individuals. To determine the error rates of these methods (Vähä & Primmer, 2006), simulated individuals of known “nonadmixed” or hybrid status (Hoban et al., 2012; Lepais et al., 2009) at a range of thresholds were analyzed. This allowed us to determine which admixture probability threshold would maximize the accuracy of assignments.

Using the simulated data, we calculated the accuracy and efficiency of the two software programs (Vähä & Primmer, 2006), testing different admixture thresholds to conclude an individual is a hybrid (Hoban et al., 2012; Lepais et al., 2009). “Efficiency” is defined as the number assigned to a category of the total number simulated in that category; low efficiency indicates that the clustering software failed to identify many individuals it attempted to assign (e.g., a missed call). “Accuracy” is defined as the number correctly assigned to a category of the total number assigned to that category, for example, the proportion that belongs there; low accuracy means that many individuals were assigned to a category incorrectly. These two values reflect erroneous omissions and inclusions, respectively. Vähä and Primmer (2006) multiply these two statistics to summarize overall “performance.” For STRUCTURE, we calculated efficiency, accuracy, and performance for thresholds from 0.86 to 0.99 (at intervals of 0.01, e.g., 0.86, 0.87), with individuals above the threshold being assigned as nonadmixed bontebok. The program NEWHYBRIDS, which produces outputs of a probability of being in one of six categories rather than one of two clusters, we tested two sets of thresholds. First, we tested a threshold of 0.50–0.99, with individuals being assigned to one of the six categories with a probability higher than the threshold. Individuals with no assignment probability above the threshold are considered “other” (unassigned). Secondly, we tested a thresholdof 0.86–0.99, in which we only assigned individuals as bontebok, blesbok, or hybrid (with hybrids being those below the threshold). The second approach is more similar to the threshold described for STRUCTURE. We calculated efficiency, accuracy, and performance for the full simulated dataset, and for a subset without the double backcross individuals, to quantify the degree to which double backcross individuals are problematic. Lastly, using simulated data, we quantified the consequences of different thresholds on the number of bontebok and hybrids removed, for thresholds between 0.86 and 0.99.

### 2.5 Spatial analysis of the dataset and management options

Using the optimized hybrid thresholds, we examined the hybridization rates at each sampling location, considering locations with at least 10 individuals per location (*n *= 76 locations) in order to test whether hybridization rates were similar across sampling locations. We tested this hypothesis as hybridization may be associated with particular anthropogenic or environmental factors and can vary across a species range (Costa et al., 2013; Hoban et al., 2012). Finally, we evaluated the management consequences of implementing a hybrid culling policy at different admixture thresholds with the goal to maximize the removal of hybrid individuals and blesbok while minimizing the removal of bontebok.

## 3 Results

### 3.1 Genetic diversity

All individuals were typed at 12 microsatellite loci, and overall, missing data were less than 2.2%. No departures from HW proportions or linkage disequilibrium were detected in each subspecies. In addition, null alleles were not observed by MICRO‐CHECKER. As previously reported by Van Wyk et al., 2013, we found that reference blesbok have significantly more variation than reference bontebok (He* *= 0.543 compared to 0.321, *p *= .025). This is partly due to near homozygosity at three markers (BB10, BB05, and BB08) in bontebok. Exclusion of these three markers still resulted in the observed lower heterozygosity levels in bontebok compared to blesbok but not significantly so (He* *= 0.422 compared to 0.531, *p *= .211).

### 3.2 Identification and classification of hybrids

**Figure 2:**
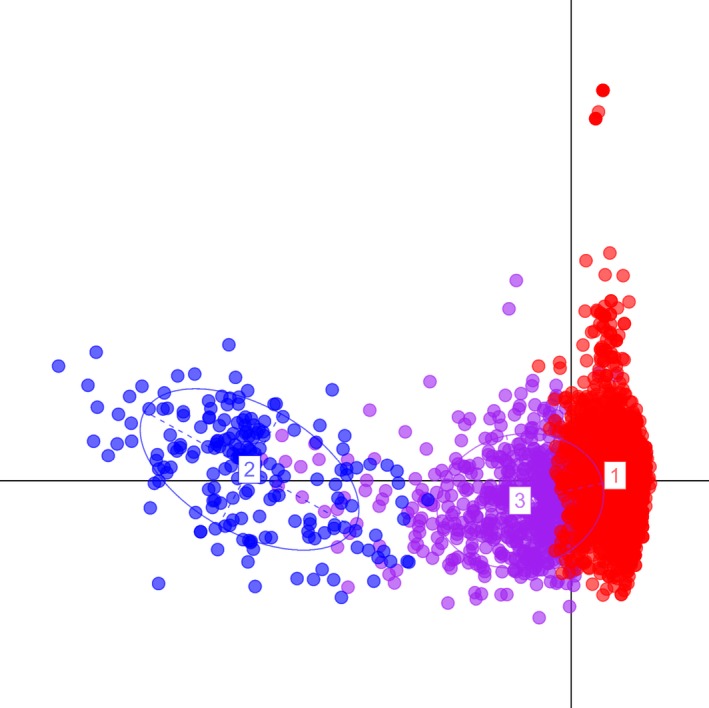
Principle component analysis (PCA) indicating the relationships among bontebok (1), blesbok (2), and hybrid (3) individuals without priori grouping

Both the species are clearly distinct on PCA, with hybrids separated between the bontebok and blesbok (Figure 2). The blesbok appears to be more scattered which may indicate higher genetic diversity. A total of 17 blesbok‐specific alleles distributed across eight loci were observed. In total, 100% of blesbok, 83.8% of hybrids (from the STRUCTURE results), and only 1.1% of bontebok had at least one blesbok‐specific allele. Thus, the method of classifying bontebok versus nonbontebok individuals based on blesbok‐specific alleles performed moderately well. In addition, four fixed alleles at two loci were observed in bontebok. Fixed alleles in bontebok did not allow clear distinction between hybrids and bontebok. While no blesbok and 96.8% of bontebok had all these alleles, 34.6% of hybrids were also fixed at the two loci known in bontebok. Retaining only individuals fixed at bontebok alleles will only eliminate 65.4% of hybrids.

**Figure 3:**
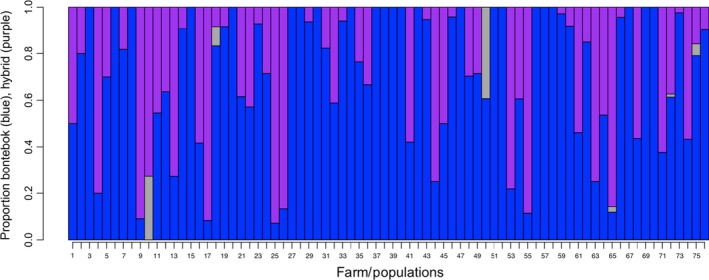
Proportion of bontebok, blesbok, and hybrids per farm (population). Each farm (population) is represented by a single vertical line, with lengths of the colored blocks proportional to the percentage of animals designated as bontebok (blue), hybrid (purple), and blesbok (gray) by the clustering analysis. Farms 1–6 includes 10 individuals, farms 7–14 includes 11 individuals, farms 15–19 includes 12 individuals, farms 20–21 includes 13 individuals, farms 22–25 includes 14 individuals, farms 26–28 includes 15 individuals, farms 29 and 30 includes 16 individuals, farms 31–35 includes 17 individuals, farms 36–39 includes 18 individuals, farms 40–43 includes 19 individuals, farm 44 includes 20 individuals, farm 45 includes 22 individuals, farm 46 includes 24 individuals, farm 47 includes 26 individuals, farm 48 includes 27 individuals, farm 49 and 50 includes 28 individuals, farm 51 includes 29 individuals, farm 52 includes 30 individuals, farm 53 includes 32 individuals, farm 54 includes 33 individuals, farms 55–57 includes 35 individuals, farms 58 and 59 includes 36 individuals, farm 60 includes 37 individuals, farm 61 includes 39 individuals, farms 62 and 63 includes 40 individuals, farm 64 includes 41 individuals, farm 65 includes 42 individuals, farm 66 includes 46 individuals, farm 67 includes 47 individuals, farm 68 includes 55 individuals, farms 69 and 70 includes 66 individuals, farm 71 includes 69 individuals, farm 72 includes 75 individuals, farm 73 includes 84 individuals, farm 74 includes 104 individuals, and farm 75 includes 158 individuals. Lastly, farm 76 includes 453 individuals collected by a single individual

Analysis of parameter settings, models, and run lengths in STRUCTURE indicated that very long runs showed superior performance compared to shorter runs (fewer individuals whose *Q*‐values changed substantially, Table 2). Comparing model settings, the uncorrelated model showed greater consistency (Table 2; also for very long runs none of the individual *Q*‐values changed by more than 0.05, for both real and simulated data). Results were highly consistent across runs (Table 3), although a small number of individuals changed status among runs, and a few individuals showed substantial change in their *Q*‐values. The underlying model has limited influence on admixture designations (Table 3). Lastly, under all parameters, confidence intervals (CI) on *Q*‐values were small: More than 50% of individual's CIs were <0.04 on either side of *Q*, more than 90% of CIs were <0.20 on either side. There was no significant relationship between CI and the percentage of missing data (linear model, *p *= .218). In regard to the program NEWHYBRIDS, very long runs and the uniform prior performed superior (Table 4) compared to the shorter runs. Choice of prior had a minimal effect on the results with high consistency across individual runs of the software (high‐consistency assignments to each category, Table 1). As with STRUCTURE, a few individuals changed status among runs. Considering these results, we chose to use very long runs and uncorrelated frequencies to generate STRUCTURE cluster membership *Q*‐values, and long runs with the uniform priors to generate NEWHYBRIDS individual hybrid category probability values, for final results. In comparing STRUCTURE and NEWHYBRIDS results, we also found relatively similar consistency in assignments to each class (Table 5). Also, the number of individuals with different assignments between the two software programs is small, close to 2%. Thus, using both methods, our unknown individuals consisted of approximately 1% blesbok, 75% bontebok, and 24% hybrid (Table 1). According to NEWHYBRIDS (Table 1), hybrids were primarily backcrossed to bontebok and F2 individuals, with some “other” (no strong assignment to one category), and no backcrosses to blesbok or F1 hybrids were identified. We found that the proportion of hybrids at each collection locality varied (Figure 3). Only 23 locations had only nonadmixed bontebok (30.2%). We also tested the hypothesis that smaller populations would have more hybrid individuals because of fewer mate choices available to breeding individuals. However, this hypothesis was not supported (*p *= .54, linear model).

**Table 1:** Number of individuals assigned to each admixture class by NEWHYBRIDS

	Blesbok	Bontebok	F1	F2	BC bontebok	BC blesbok	Other
Run 1, uniform	184	2,113	–	67	54	–	558
Run 2, uniform	184	2,106	–	59	60	–	567
Run 3, uniform	184	2,102	–	68	50	–	572
Run 1, Jeffreys	183	2,099	–	83	60	–	551
Run 2, Jeffreys	184	2,097	–	72	68	–	555
Run 3, Jeffreys	183	2,097	–	84	59	–	553

**Table 2:** Mean number of individuals whose *Q*‐values increased or decreased by more than 0.05 when compared to another run (mean of 4 runs)

Run length	Correlated	Uncorrelated
Short	53	15
Long	57	4
Very long	39	0

**Table 3:** Number of individuals assigned to each admixture class by STRUCTURE

Run	Blesbok	Bontebok	Other
Run 1, uncorrelated	184	2,125	667
Run 2, uncorrelated	184	2,127	665
Run 3, uncorrelated	184	2,127	665
Run 1, correlated	184	2,082	710
Run 2, correlated	184	2,065	727
Run 3, correlated	184	2,082	710

**Table 4:** Mean number of individuals whose assigned identity changed among runs (mean of 3 runs)

Run	Uniform	Jeffrey's
Medium	46	97
Very long	21	26

**Table 5:** Composition of real dataset, according to STRUCTURE and NEWHYBRIDS at “best” final settings (see text for definition of final settings)

Approach	Blesbok	Bontebok	Hybrid
Structure (unknown + reference individuals)	184	2,127	665
NEWHYBRIDS (unknown + reference individuals)	184	2,113	679
Structure (only unknown individuals)	29	2,051	657
NEWHYBRIDS (only unknown individuals)	29	2,038	670

### 3.3 Analysis of simulated data

Using simulated data, we determined the threshold values that could be applied to assign individuals as nonadmixed or admixed in an empirical dataset. We tested the efficiency and accuracy of the two Bayesian methods using thresholds ranging from 0.85 to 0.99 (Tables S1 and S2). For assignment to each nonadmixed species, efficiency was maximized at low thresholds, while accuracy was maximized at high thresholds, while for hybrids, efficiency was maximized at high thresholds and accuracy was maximized at low thresholds (for both programs, see Table S1). A subjectively determined balance appears to be achieved at a threshold between 0.90 and 0.95 for STRUCTURE. Double backcrosses individuals decreased both the accuracy and the efficiency of both methods, as they were difficult to detect, and will change the recommended threshold. For example, with NEWHYBRIDS, maximum accuracy of identifying nonadmixed individuals was achieved with a threshold between 0.8 and 0.88 without double backcross, but 0.93 and 0.97 with them. The two programs had similar results regarding accuracy, efficiency, and performance.

**Figure 4:**
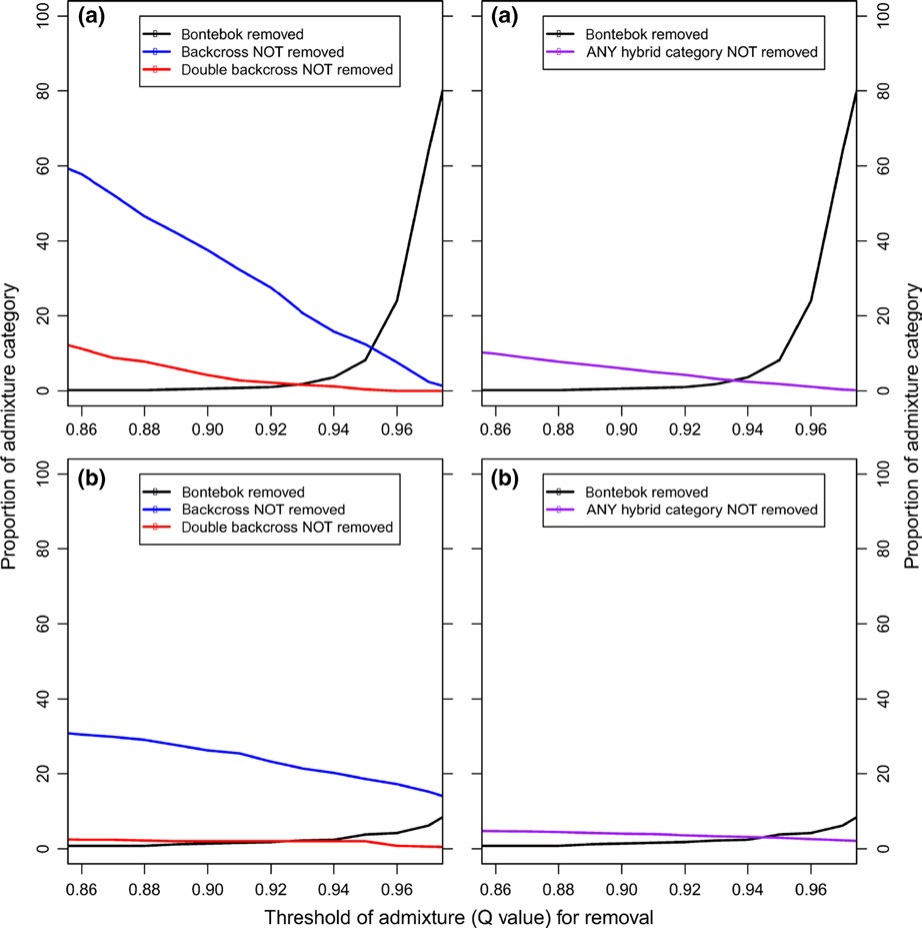
The proportion of simulated bontebok animals wrongly removed (black line) and simulated hybrid animals wrongly not removed (coloured lines) for STRUCTURE (a) and NEWHYBRIDS (b)

**Figure 5:**
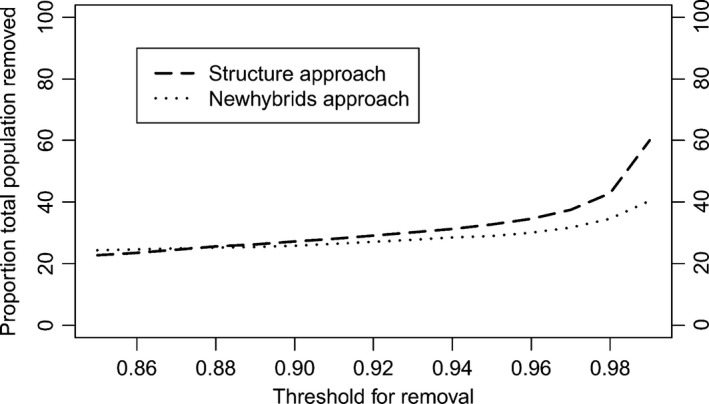
The proportion of the real population of animals that will be removed at different thresholds

Lastly, we tested the threshold for removal on the simulated data (Figure 4a,b) to quantify the proportion of bontebok (black line) and the proportion of the other categories that would be incorrectly assigned (colored lines). As the threshold increased, especially beyond 0.94 for STRUCTURE (Figure 4a), a large proportion of nonadmixed bontebok were incorrectly removed, to capture the backcrosses and double backcrosses. Similar patterns were seen for both programs, with the main difference being that for NEWHYBRIDS (Figure 4b) fewer bontebok were removed, but slightly more hybrids were included at higher thresholds. Note that NEWHYBRIDS performs poor at assigning individuals to each particular hybrid category, for example, assigning an individual that really is an F2 to the F2 category rather than classifying as a general “hybrid” category (data not shown). We then applied the threshold to our actual dataset to quantify how many individuals will be removed in a culling program to retain only nonadmixed bontebok (Figure 5). A low‐to‐moderate threshold (0.85–0.92) implies that approximately 20%–30% of all living animals will need to be culled (with similar results for each software), while at the highest thresholds between 40% and 60% of animals will be culled (depending on the software used).

## 4 Discussion

In this study, we examined hybridization rates between bontebok and blesbok in nearly 3,000 animals sampled from across South Africa. Overall, approximately 25% of nonreference animals are hybrids, which is slightly lower than previously estimated in a smaller study (33% estimated from 121 animals; van Wyk et al. 2013). Importantly, admixed individuals were found in two‐thirds of the locations included here. Thus, few populations on private land can be considered as nonadmixed. As the game breeding and hunting industry advances, translocation and hybridization (intentional and unintentional) rates will likely increase. Unless hybrids are removed, this increase combined with the current substantial overall numbers of hybrids and the higher number of locations with hybrids could ultimately result in swamping of the native gene pool as has been reported in other species (Huxel, 1999; Vaz Pinto et al., 2016).

Both the admixture software programs STRUCTURE and NEWHYBRIDS performed equally well and provided similar hybridization rates (Table 5). Other settings such as the model chosen affect the designation of a very small number of individual animals (Tables 1, 3, 4). Thus, the uncertainty regarding any given individual animal's assignment is relatively low. Overall, our conclusion about hybridization rates is robust. We also found that blesbok private alleles can be used to assist in identifying hybrids (classifying nearly 84% of hybrids); however, the probability‐based approaches in STRUCTURE and NEWHYBRIDS had a higher efficiency and accuracy in detecting hybrid individuals (>95%, depending on thresholds selected).

We identified clear trade‐offs in accuracy and efficiency of hybrid identification. From a conservation perspective, for this rare subspecies, it is more desirable to have high efficiency with respect to hybrids and high accuracy in the case of nonadmixed bontebok. Thus, a semi‐conservative threshold (0.90 using STRUCTURE; note that different thresholds apply to each of the programs—different thresholds should be used for NEWHYBRIDS) is preferable to ensure that nonadmixed bontebok are not mistakenly designated as hybrids. In this case, it would be advisable not to cull hybrids to preserve native individuals and their native gene pool. Thresholds at or above 0.94 (for STRUCTURE) were deemed unacceptable due to high proportions of nonadmixed bontebok that would be accidentally removed (e.g., 8.2% for 0.94 threshold and 64% for 0.97 threshold). Removing a larger proportion of nonadmixed bontebok would negatively affect the genetic and demographic viability of the subspecies and might lead to “genetic erosion.” It is important to observe that different thresholds also influence the scope of the culling management implementation. Removing more individuals would require a larger number of resources. The number of animals to be removed via management could vary from 20% to 60% of the existing census. It should be noted that double backcrosses to bontebok individuals are quite difficult to detect, and detecting them requires higher *Q*‐value thresholds (which would remove more bontebok). Indeed, only 62.4% of double backcrosses will be removed with a threshold of 0.90 that was chosen. If the bontebok population was not at such a low number already, a higher threshold and thus removal of more bontebok may be acceptable. It is acknowledged that identification of more complex hybrid categories (e.g., crosses between F1 and backcrosses), as well as further generations of crossing may be possible in the future by using more markers (e.g., SNPs). Developing more markers to achieve such resolution is recommended. A formal framework to incorporate data from *Q*‐values, species‐specific alleles, and morphology would be imperative to ensure the long‐term survival of bontebok.

The results from this study indicate that hybridization in these subspecies is historical and that hybridization is not occurring between nonadmixed bontebok and nonadmixed blesbok but rather between nonadmixed bontebok/nonadmixed blesbok and hybrids, as no F1 hybrids were identified in this study using the method of NEWHYBRIDS (which assigns individuals to different hybrid categories rather than the *Q*‐values of STRUCTURE). Interestingly, a study of polecats in Britain also found no F1 hybrids in a survey of 345 animals; however, extensive admixture rates were identified (Costa et al., 2013) as observed in this study. In addition, we identified no backcrosses to blesbok. One possible explanation is that management strategies on private land include backcrossing with bontebok in order to obtain nonadmixed bontebok populations.

Bontebok was a conservation flagship species for the former (pre‐1994) Cape Province and strict regulatory measures to protect bontebok from deliberate and/or accidental hybridization with imported blesbok were implemented in the late 1980s. These measures included the demarcation of natural distributional ranges based on magisterial (administrative district) boundaries to inform translocation through permits. The limited size and availability of habitat within the “natural distributional range” led to the demarcation of an additional area, an “extended distribution range,” to which bontebok may be translocated to enable population expansion. The consolidated area was buffered with a region where no introductions of either bontebok or blesbok would be permitted in order to secure the bontebok population through separation. Bontebok populations on private land were assessed using a computer‐based method developed to distinguish between nonadmixed bontebok, blesbok, and hybrid populations based on the measurements of the rump patch taken from photographs as per the method described by Fabricius et al. (1989). Populations comprised of nonadmixed bontebok were certified, and bontebok purity certificates were issued to land owners as incentives to promote population expansion. These certificates are required for any imports of bontebok hunting trophies into the United States. Nonadmixed bontebok individuals have become a novelty, and the demand for stocking of this subspecies outside its indigenous range has increased substantially. Game species have been legally imported and exported onto private land, outside the indigenous range of the subspecies, resulting in an increase in the demand for bontebok sourced from its indigenous range.

To preserve remaining native bontebok populations, policy and management need to be implemented quickly. Our molecular data and modeling results have helped in this effort. The Western Cape Provincial Conservation Agency, CapeNature, developed the Bontebok Conservation, Translocation and Utilization Policy (BCTUP, 2014) as a regulatory mechanism to implement the genetic test described in this article to advise on permitting translocations. The result from this research was directly used in order to establish the optimal threshold of admixture in this policy. All bontebok that are translocated must be tested and permanently marked (microchipped). Test results will be stored in a centralized database at the National Zoological Gardens of South Africa. BCTUP contains a collection protocol specifically aimed at the implementation of a forensic sampling procedure and the maintenance of custody samples. At present, identified hybrids must be kept in isolation, they may not be translocated alive, and must be culled. We hope that our quantitative assessment of hybrids in this system is an exemplar for future studies of hybridization in southern Africa, and furthers the development of science‐based, quantitative conservation policies.

## Conflict of Interest

None declared.

## References

[ece32595-bib-0001] Allendorf, F. W. , Leary, R. F. , Spruell, P. , & Wenburg, J. K. (2001). The problems with hybrids: Setting conservation guidelines. Trends in Ecology and Evolution, 16, 613–622.

[ece32595-bib-0002] Allendorf, F. W. , & Luikart, G. (2007). Conservation and the genetics of populations. Oxford, UK: John Wiley & Sons.

[ece32595-bib-0003] Allendorf, F. W. , Luikart, G. , & Aitken, S. N. (2013). Conservation and the genetics of populations, 2nd ed. New York: Wiley‐Blackwell.

[ece32595-bib-0004] Anderson, E. C. , & Thompson, E. A. (2002). A model‐based method for identifying species hybrids using multilocus data. Genetics, 160, 1217–1229. 11901135 10.1093/genetics/160.3.1217 PMC1462008

[ece32595-bib-0005] Bigalke, R. (1955). The bontebok *Damaliscus pygargus* with a special reference to its history and preservation. Fauna and Flora, 6, 95–116.

[ece32595-bib-0100] Birss, C. , van Deventer, J. D. , Hignett, D. L. , Brown, C. , Gildenhuys, P. , & Kleinhans, D. (2014). CapeNature Bontebok Conservation, Translocation and Utilization Policy (BCTUP). Version1 Operational Guideline.

[ece32595-bib-0006] Cordingley, J. E. , Sundaresan, S. R. , Fischhoff, I. R. , et al. (2009). Is the endangered Grevy's zebra threatened by hybridization? Animal Conservation, 12, 505–513.

[ece32595-bib-0007] Costa, M. , Fernandes, C. , Birks, J. D. , Kitchener, A. C. , Santos‐Reis, M. , & Bruford, M. W. (2013). The genetic legacy of the 19th‐century decline of the British polecat: Evidence for extensive introgression from feral ferrets. Molecular Ecology, 22, 5130–5147. 24050727 10.1111/mec.12456

[ece32595-bib-0008] Cullingham, C. I. , Cooke, J. E. K. , Dang, S. , et al. (2011). Mountain pine beetle host‐range expansion threatens the boreal forest. Molecular Ecology, 20, 2157–2171. 21457381 10.1111/j.1365-294X.2011.05086.x PMC3116149

[ece32595-bib-0009] Dalton, D. L. , Tordiffe, A. , Luther, I. , et al. (2014). Interspecific hybridization between greater kudu and nyala. Genetica, 142, 265–271. 24906427 10.1007/s10709-014-9772-7

[ece32595-bib-0010] Essop, M. F. , Lloyd, P. H. , Van Hensbergen, H. J. , & Harley, E. H. (1991). Estimation of the genetic distance between bontebok and blesbok using mitochondrial DNA. South African Journal of Science, 87, 271–273.

[ece32595-bib-0011] Fabricius, C. , van Hensbergen, H. , & Zucchini, W. (1989). A discriminant function for identifying hybrid bontebok x blesbok populations. African Journal of Wildlife Research, 19, 61–66.

[ece32595-bib-0012] Gelman, A. (2009). Bayes, Jeffreys, prior distributions and the philosophy of statistics. Statistical Science, 24, 176–178.

[ece32595-bib-0013] Green, W. C. H. , & Rothstein, A. (1998). Translocation. Hybridization, and the Endangered Black‐Faced Impala, 12, 475–480.

[ece32595-bib-0014] Grobler, J. P. , Rushworth, I. , Brink, J. S. , et al. (2011). Management of hybridization in an endemic species: Decision making in the face of imperfect information in the case of the black wildebeest‐Connochaetes gnou. European Journal of Wildlife Research, 57, 997–1006.

[ece32595-bib-0015] Hoban, S. (2014). An overview of the utility of population simulation software in molecular ecology. Molecular Ecology, 23, 2383–2401. 24689878 10.1111/mec.12741

[ece32595-bib-0016] Hoban, S. M. , Hauffe, H. C. , Pérez‐Espona, S. , et al. (2013). Bringing genetic diversity to the forefront of conservation policy and management. Conservation Genetics Resources, 5, 593–598.

[ece32595-bib-0017] Hoban, S. M. , McCleary, T. S. , Schlarbaum, S. E. , Anagnostakis, S. L. , & Romero‐Severson, J. (2012). Human‐impacted landscapes facilitate hybridization between a native and an introduced tree. Evolutionary Applications, 5, 720–731. 23144658 10.1111/j.1752-4571.2012.00250.x PMC3492897

[ece32595-bib-0018] Hubisz, M. J. , Falush, D. , Stephens, M. , & Pritchard, J. K. (2009). Inferring weak population structure with the assistance of sample group information. Molecular Ecology Resources, 9, 1322–1332. 21564903 10.1111/j.1755-0998.2009.02591.x PMC3518025

[ece32595-bib-0019] Huxel, G. R. (1999). Rapid displacement of native species by invasive species: Effects of hybridization. Biological Conservation, 89, 143–152.

[ece32595-bib-0020] Jombart, T. (2008). adegenet: A R package for the multivariate analysis of genetic markers. Bioinformatics (Oxford, England), 24, 1403–1405. 10.1093/bioinformatics/btn129 18397895

[ece32595-bib-0021] Jombart, T. , & Ahmed, I. (2011). adegenet 1.3‐1: New tools for the analysis of genome‐wide SNP data. Bioinformatics (Oxford, England), 27, 3070–3071. 10.1093/bioinformatics/btr521 PMC3198581 21926124

[ece32595-bib-0022] Laikre, L. , Schwartz, M. K. , Waples, R. S. , & Ryman, N. (2010). Compromising genetic diversity in the wild: Unmonitored large‐scale release of plants and animals. Trends in Ecology & Evolution, 25, 520–529. 20688414 10.1016/j.tree.2010.06.013

[ece32595-bib-0023] Lepais, O. , Petit, R. J. , Guichoux, E. , et al. (2009). Species relative abundance and direction of introgression in oaks. Molecular Ecology, 18, 2228–2242. 19302359 10.1111/j.1365-294X.2009.04137.x

[ece32595-bib-0024] Levin, D. A. , Francisco‐Ortega, J. , & Jansen, R. K. (1996). Hybridization and the extinction of rare plant species. Conservation Biology, 10, 10–16.

[ece32595-bib-0025] Lloyd, P. , & David, J. (2008). Damaliscus pygargus. The IUCN Red List of Threatened Species 2008: e.T30208A9530977. Retrieved from http://dx.doi.org/10.2305/IUCN.UK.2008.RLTS.T30208A9530977.en.

[ece32595-bib-0026] Malukiewicz, J. , Boere, V. , Fuzessy, L. F. , et al. (2014). Hybridization effects and genetic diversity of the common and black‐tufted marmoset (*Callithrix jacchus* and *Callithrix penicillata*) mitochondrial control region. American Journal of Physical Anthropology, 155, 522–536. 25186076 10.1002/ajpa.22605

[ece32595-bib-0027] Metcalf, J. L. , Siegle, M. R. , & Martin, A. P. (2008). Hybridization dynamics between Colorado's native cutthroat trout and introduced rainbow trout. The Journal of Heredity, 99, 149–156. 18238784 10.1093/jhered/esm118

[ece32595-bib-0028] Nielsen, E. E. , Bach, L. A. , & Kotlicki, P. (2006). HYBRIDLAB (version 1.0): A program for generating simulated hybrids from population samples. Molecular Ecology Notes, 6, 971–973.

[ece32595-bib-0029] Park, S. (2001). The Excel microsatellite toolkit. Trypanotolerance in west African cattle and the population genetic effects of selection. Ph.D thesis, University of Dublin, Dublin, Ireland.

[ece32595-bib-0030] Peakall, R. , & Smouse, P. E. (2006). GenAlEx 6: genetic analysis in Excel. Population genetic software for teaching and research. Molecular Ecology Notes, 6, 288–295. 10.1093/bioinformatics/bts460 PMC3463245 22820204

[ece32595-bib-0031] Peakall, R. , & Smouse, P. E. (2012). GenAlEx 6.5: Genetic analysis in Excel. Population genetic software for teaching and research—An update. Bioinformatics (Oxford, England), 28, 2537–2539. 10.1093/bioinformatics/bts460 PMC3463245 22820204

[ece32595-bib-0032] Piett, S. , Hager, H. A. , & Gerrard, C. (2015). Characteristics for evaluating the conservation value of species hybrids. Biodiversity and Conservation, 24, 1931–1955.

[ece32595-bib-0033] Pritchard, J. K. , Stephens, M. , & Donnelly, P. (2000). Inference of population structure using multilocus genotype data. Genetics, 155, 945–959. 10835412 10.1093/genetics/155.2.945 PMC1461096

[ece32595-bib-0034] Raymond, M. , & Rousset, F. (1995). An exact test for population differenciation. Evolution, 49(6), 1280–1283. 10.1111/j.1558-5646.1995.tb04456.x 28568523

[ece32595-bib-0035] Rhymer, J. M. , & Simberloff, D. (1996). Extinction by hybridization and introgression. Annual Review of Ecology and Systematics, 27, 83–109.

[ece32595-bib-0036] Sanz, N. , Araguas, R. M. , Fernández, R. , Vera, M. , & García‐Marín, J.‐L. (2008). Efficiency of markers and methods for detecting hybrids and introgression in stocked populations. Conservation Genetics, 10, 225–236.

[ece32595-bib-0037] Schwartz, M. K. , Luikart, G. , & Waples, R. S. (2007). Genetic monitoring as a promising tool for conservation and management. Trends in Ecology & Evolution, 22, 25–33. 16962204 10.1016/j.tree.2006.08.009

[ece32595-bib-0038] Skead, C. (1980). Historical mammal incidence in the Cape Province 1. Cape Town: Department of Nature and Environmental Conservation.

[ece32595-bib-0039] Skinner, J. D. , & Smithers, R. (1990). Alcelaphini The mammals of Southern African subregion (pp. 612–634). Pretoria: University of Pretoria Press.

[ece32595-bib-0040] Spear, D. , & Chown, S. L. (2009). The extent and impacts of ungulate translocations: South Africa in a global context. Biological Conservation, 142, 353–363.

[ece32595-bib-0041] Stephens, D. , Wilton, A. N. , Fleming, P. J. S. , & Berry, O. (2015). Death by sex in an Australian icon: A continent‐wide survey reveals extensive hybridization between dingoes and domestic dogs. Molecular Ecology, 24, 5643–5656. 26514639 10.1111/mec.13416

[ece32595-bib-0042] Todesco, M. , Pascual, M. A. , Owens, G. L. , et al. (2016). Hybridization and extinction. Evolutionary Applications, 9, 892–908. doi:10.1111/eva.12367 27468307 10.1111/eva.12367 PMC4947151

[ece32595-bib-0043] Vähä, J. P. , & Primmer, C. R. (2006). Efficiency of model‐based Bayesian methods for detecting hybrid individuals under different hybridization scenarios and with different numbers of loci. Molecular Ecology, 15, 63–72. 16367830 10.1111/j.1365-294X.2005.02773.x

[ece32595-bib-0044] Valbuena‐Carabaña, M. , González‐Martínez, S. C. , Hardy, O. J. , & Gil, L. (2007). Fine‐scale spatial genetic structure in mixed oak stands with different levels of hybridization. Molecular Ecology, 16, 1207–1219. 17391407 10.1111/j.1365-294X.2007.03231.x

[ece32595-bib-0045] Van der Merwe, N. (1986). Die bontebok. Koedoe, 11, 161–168.

[ece32595-bib-0046] Van der Walt, J. M. , Nel, L. H. , & Hoelzel, A. R. (2001). Characterization of major histocompatibility complex DRB diversity in the endemic South African antelope *Damaliscus pygargus*: A comparison in two subspecies with different demographic histories. Molecular Ecology, 10, 1679–1688. 11472536 10.1046/j.0962-1083.2001.01321.x

[ece32595-bib-0047] Van Oosterhout, C. , Hutchinson, W. F. , Wills, D. P. M. , & Shipley, P. (2004). MICRO‐CHECKER: Software for identifying and correcting genotyping errors in microsatellite data. Molecular Ecology Notes, 4, 535–538.

[ece32595-bib-0048] Van Wyk, A. M. , Kotzé, A. , Randi, E. , & Dalton, D. L. (2013). A hybrid dilemma: A molecular investigation of South African bontebok (*Damaliscus pygargus pygargus*) and blesbok (*Damaliscus pygargus phillipsi)* . Conservation Genetics, 14, 589–599.

[ece32595-bib-0049] Vaz Pinto, P. , Beja, P. , Ferrand, N. , & Godinho, R. (2016). Hybridization following population collapse in a critically endangered antelope. Scientific Reports, 6, 18788. 26732144 10.1038/srep18788 PMC4702127

[ece32595-bib-0050] Vrba, E. S. (1979). Phylogenetic analysis and classification of fossil and recent Alcelaphini Mammalia: Bovidae. Biological Journal of the Linnean Society, 11, 207–228.

[ece32595-bib-0051] Wolf, D. E. , Takebayashi, N. , & Rieseberg, L. H. (2001). Predicting the risk of extinction through hybridization. Conservation Biology, 15, 1039–1053.

